# Kidney function and nephrotoxic drug use among older home-dwelling persons with or without diabetes in Finland

**DOI:** 10.1186/s12882-020-1684-4

**Published:** 2020-01-10

**Authors:** Marjo Heinjoki, Merja Karjalainen, Juha Saltevo, Miia Tiihonen, Maija Haanpää, Hannu Kautiainen, Pekka Mäntyselkä

**Affiliations:** 1 School of Pharmacy, University of Eastern Finland, P.O. BOX 1627, FI-70211, Kuopio, Finland; 2 Institute of Public Health and Clinical Nutrition, General Practice, University of Eastern Finland, Kuopio, Finland; 3 Inner Savo Health Center, Suonenjoki, Finland; 4 Central Finland Central Hospital, Jyväskylä, Finland; 5 Ilmarinen Mutual Pension Insurance Company, Helsinki, Finland; 6 Department of Neurosurgery, Helsinki University Hospital, Helsinki, Finland; 7 Unit of Primary Health Care, Helsinki University Central Hospital, Helsinki, Finland; 8 Primary Health Care Unit, Kuopio University Hospital, Kuopio, Finland

**Keywords:** Diabetes mellitus, eGFR, Older people, Nephrotoxic drugs

## Abstract

**Background:**

Due to these changes in kidney function, aging kidneys are more prone to drug-induced impairments in renal properties. Diabetes has been associated with the declined kidney function and an elevated risk of renal failure. The aim of this study is to compare kidney function and potentially nephrotoxic drug use among home-dwelling older persons with or without diabetes.

**Methods:**

A total of 259 persons with and 259 persons without diabetes and aged ≥65 years were randomly selected to participate in a health examination with complete data gathered from 363 individuals (187 with diabetes and 176 without diabetes). The estimated glomerular filtration rate (eGFR) was calculated using CKD-EPI equation. Each participant was categorized based on the nephrotoxic profile of their medications.

**Results:**

There were no differences in mean eGFR values (77.5 ± 18.8 vs. 80.5 ± 14.8 ml/min/1.73m^2^, *p* = 0.089) or in the proportion of participants with eGFR < 60 ml/min/1.73m^2^ among persons with diabetes (16% vs. 10%, *p* = 0.070), compared to persons without diabetes. Potentially nephrotoxic drug use was similar between the groups. The mean number of potentially nephrotoxic drugs was 1.06 ± 0.88 in those with and 0.97 ± 1.05 in those without diabetes (*p* = 0.39).

**Conclusions:**

The kidney function of older persons with diabetes does not differ from that of older persons without diabetes and furthermore potentially nephrotoxic drug use seem to play only a minor role in the decline in kidney function among home-dwelling persons in the Inner-Savo district.

## Background

The global prevalence of diabetes increased by 30.6% during the period 2005–2015 [1]. In 2030, it is estimated that 552 million people will have received a diagnosis of diabetes and the rise is expected to be highest in the oldest age groups [2]. In global terms, in conjunction with hypertension, diabetes is the most common cause of chronic kidney disease [3]. It has been associated with a higher rate in the decline of kidney function and an elevated risk of renal failure [4–8].

Even healthy aging causes structural and functional changes in the kidneys and the number of nephrons declines with the age [9]. For example, individuals aged 70 to 75 years have 48% fewer nephrons when compared to persons aged 18 to 29 years [10]. The loss of nephrons leads to a decline in the glomerular filtration rate (GFR). Comorbidities such as hypertension and diabetes combined with normal aging can lead to additional nephron losses and nephrosclerosis as well as larger glomeruli [9].

Due to these changes in kidney function, aging kidneys are more prone to drug-induced impairments in renal properties [11–13]. There are reports that a number, from 6% [14] to 19% [15], of community-acquired acute kidney injuries (AKIs) were caused by nephrotoxic drugs. There are changes in the pharmacokinetics of several drugs as a result of the altered kidney function and therefore kidney function measurements are strongly recommended when prescribing medications to older persons [11–13, 16].

## Method

### Aim of the study

The societal burden of diabetes is rising, especially in the oldest age groups but only a few studies have focused on the kidney function of older persons with diabetes and with their use of nephrotoxic drugs [6, 8, 17–21]. Hence, the aims of this study were to compare kidney function and use of potentially nephrotoxic drugs occurring among older home-dwelling persons with or without diabetes.

### Study population

The research data of this cross-sectional study is part of the data collected for the Inner-Savo Diabetes Mellitus research (ISDM). The study population (*N* = 3093) consisted of home-dwelling people aged 65 years and older living in the Inner-Savo region in Finland. The electronic patient records of the Inner-Savo primary health care system were used in order to identify persons with a diagnosis of diabetes (*n* = 540, diagnostic codes of E10; type 1 diabetes mellitus and E11; type 2 diabetes mellitus) in accordance with International Classification of Diseases (ICD-10) [22]. Two age and gender matching controls were selected for each person diagnosed with diabetes using primary health care system. In autumn 2015, health questionnaires were sent to a total of 1417 persons, i.e. 527 persons with diabetes and 890 persons without diabetes. In all, 518 of the 1084 questionnaire respondents (response rate 76.5%), of which 259 with and 259 without diabetes, were randomly selected to participate in a health examination. Exclusion criteria included being permanently bedridden and hospitalized along with those patients suffering from the terminal stages of cancer or other terminal illnesses. Ultimately, complete data was gathered from 187 persons with diabetes (type 1 diabetes *n* = 5, type 2 diabetes *n* = 182) and 176 persons without diabetes.

### Measurements and tools

The standardized health examinations were conducted by one physician (MK). Weight and height and waist circumference were measured and body mass index (BMI) was calculated (weight/ height^2^) for each participant. Orthostatic blood pressure was measured after resting in a supine position for 10 min. Orthostatic blood pressure was measured in the lying, sitting and standing positions at times of one and three minutes by using an automated blood pressure monitor [23]. Blood pressure was measured in the sitting position at the time of one minute.

Routine laboratory tests were analyzed by Eastern Finland Laboratory Centre (ISLAB). ISLAB has been licensed by the Finnish Accreditation Service. Liver, kidney and thyroid gland function along with blood glucose, lipid levels and inflammation marker were determined (P-ALAT, P-Krea, P-Alb, U-Alb, fPGluk, B-HbA1C, fP-Kol, fP-Kol-HDL, fP-Kol-LDL, fP-Trigly, S-CRP). Comorbidities and the most common chronic diseases were verified by the physician from the electronic patient records and the total number of comorbidities was calculated. Six comorbidities were verified; (1) ischemic heart disease, (2) heart failure, (3) arterial fibrillation and flutter, (4) hypertensive disease, (5) cerebrovascular diseases, and (6) diseases of arteries, arterioles and capillaries.

The estimated glomerular filtration rate (eGFR) was calculated by using Chronic Kidney Disease Epidemiology Collaboration (CKD-EPI) –equation [24]. The calculated value was based on the participant’s plasma creatinine concentration, age and gender. The eGFR was used to evaluate each participant’s kidney function. CKD-EPI has been found to have good accuracy also when assessing the eGFR in obese people [25]. Additionally, eGFR with Cockcroft-Gault equation was calculated to evaluate kidney function observing the effect of weight of the participants [26].

Renbase® database was used to determine detailed drug dosing recommendations for different degrees of renal failure. Renbase® was developed by Medbase Ltd. [27]. The drug dosing recommendations were classified into four categories; A to D; the classification is presented in Table 1. In addition, the degree of renal failure is subdivided into four categories; mild to end-stage renal failure (mild: 90–60 ml/min/1.73m^2^, moderate: 60–30 ml/min/1.73m^2^, severe: 30–15 ml/min/1.73m^2^, end-stage: < 15 ml/min/1.73m^2^). The classification of renal failure is consistent with the European Medicines Agency (EMA) classification. Renbase® includes recommendations of drug dosing and safety as well as further information about the pharmacokinetics and nephrotoxicity of the drug.

**Table 1:** Classification categories and definitions in Pharao® and Renbase® [8–10]

Classification	Definition	
	Pharao	Renbase
A	No known pharmacological or clinical basis for an increased risk.	No need for dosage modification.
B	There is a somewhat increased risk.	The information is not available or the recommendation is estimated based on the pharmacokinetic characteristics of the substance.
C	There is a moderately increased risk.	Modification of the dose or dosage interval is needed.
D	There is a high risk.	The use should be avoided.
0	No pharmacological effect.	–
1	A mild pharmacological effect.	–
2	A moderate pharmacological effect.	–
3	A strong pharmacological effect.	–

The drug use was collected by physician during health examinations. The Pharmacological Risk Assessment Online system (Pharao®) is a tool which can identify the number of clinically significant drug adverse effect profiles; in this research project, it was used to identify potentially nephrotoxic drugs (Additional file 1: Table S1). The database was developed by co-operation between experts from Sweden and Finland [28]. Pharao® is a database listing the drug adverse effects developed by the working group [29]. It contains nine general and severe adverse effects of drugs and estimates overall risk scores in a scale of zero to three (Table 1). General and severe adverse effects include nephrotoxicity, QT prolongation/arrhythmia, seizures, sedation, bleeding, orthostatic hypotension, constipation, serotonergic and anticholinergic side effects.

### Ethics approval

Each of the participants gave consent to take part in this study and to allow access to personal data process by signing an informed consent form. The Research Ethics Committee of the Northern Savo Hospital District gave an approving statement for the study.

### Statistical analysis

Continuous variables were analyzed by using either a t-test or a bootstrap type t-test, and categorical variables were compared using chi-square or Fisher’s exact test where appropriate. The bootstrap (10,000 replications) method was used when the theoretical distribution of the test statistics were unknown or in the case of a violation of the assumptions (e.g. non-normality). The normality of the variables was tested by using the Shapiro-Wilk W test. The Stata 15.1, StataCorp LP (College Station, TX, USA) statistical package was used for the analysis.

## Results

A total of 363 home-dwelling persons participated in the health examination and complete data was gathered from 187 persons with and 176 persons without diabetes. The mean age of the participants was 74 years; this was identical in both groups. The proportion of females varied between the groups, 34% (*n* = 60) of the participants without diabetes and 49% (*n* = 92) of those with diabetes were females. The clinical characteristics of the participants are presented in Table 2. Persons with diabetes had a higher body mass index and a larger waist (*p* < 0.001). Systolic and diastolic blood pressures were higher among persons without diabetes (*p* = 0.05 and *p* = 0.039). Smoking was more common in persons without diabetes (*p* = 0.021).

**Table 2:** Clinical characteristics of the participants with or without diabetes

	No diabetes *N* = 176	Diabetes *N* = 187	*P*-value
Physiological factors, mean (SD)			
Body mass index, kg/m^2^	28 (5)	31 (6)	< 0.001
Waist, cm			
Male	97 (12)	107 (14)	< 0.001
Female	94 (13)	104 (15)	< 0.001
Blood pressure mmHg, mean (SD)			
Systolic	156 (22)	151 (22)	0.050
Diastolic	90 (12)	87 (11)	0.039
Smoking, *n* (%)	24 (14)	12 (6)	0.021
Comorbidities, *n* (%)			
Ischemic heart disease	29 (16)	44 (24)	0.094
Heart failure	3 (2)	4 (2)	0.76
Arterial fibrillation and flutter	22 (12)	32 (17)	0.22
Hypertensive disease	99 (56)	126 (67)	0.029
Cerebrovascular diseases	6 (4)	4 (2)	0.46
Disease of arteries, arterioles and capillaries	6 (3)	5 (3)	0.68
Laboratory values, mean (SD)			
Cholesterol, mmol/l			
Total	4.91 (1.01)	4.59 (1.13)	0.005
High-density lipoprotein	1.56 (0.44)	1.38 (0.42)	< 0.001
Low-density lipoprotein	3.00 (0.84)	2.70 (1.01)	0.002
Total triglycerides, mmol/l	1.14 (0.51)	1.56 (0.73)	< 0.001
Fasting plasma glucose, mmol/l	6.15 (3.75)	7.78 (2.41)	< 0.001
HbA1c, mmol/l	37.4 (3.5)	49.3 (13.6)	< 0.001
ALAT, U/l	22.9 (10.8)	26.8 (17.3)	0.099
P-Alb, g/l	40.4 (3.1)	39.9 (3.7)	0.16
U-Alb, mg/l	4.5 (11.9)	11.3 (38.0)	0.11
CRP, mg/l	2.2 (3.7)	3.4 (6.5)	0.034
Glomerular filtration rate			
CKD-EPI, ml/min/1.73m^2^, mean (SD)	80.5 (14.8)	77.5 (18.8)	0.089
CKD-EPI, < 60 ml/min/1.73m^2^, n (%)	17 (10)	30 (16)	0.070

*SD* standard deviation, *HbA1c* glycated hemoglobin, *ALAT* Alanine aminotransferase, *P-Alb* plasma albumin, *U-Alb* urine albumin, *CRP* C-reactive protein, *CKD-EPI* Chronic Kidney Disease Epidemiology Collaboration equation for estimated glomerular filtration rate

There was a statistically significant difference in the blood lipid profiles between the groups. Persons without diabetes had higher levels of total cholesterol, high-density and low-density lipoprotein (*p* = 0.005, *p* < 0.001, *p* = 0.002). The total triglyceride level was higher among persons with diabetes (p < 0.001). Furthermore, persons with diabetes had a significantly higher C-reactive protein value (*p* = 0.034). In both groups, the most common comorbidity was hypertensive disease; this occurred more frequently among persons diagnosed with diabetes (*p* = 0.029).

**Fig. 1:**
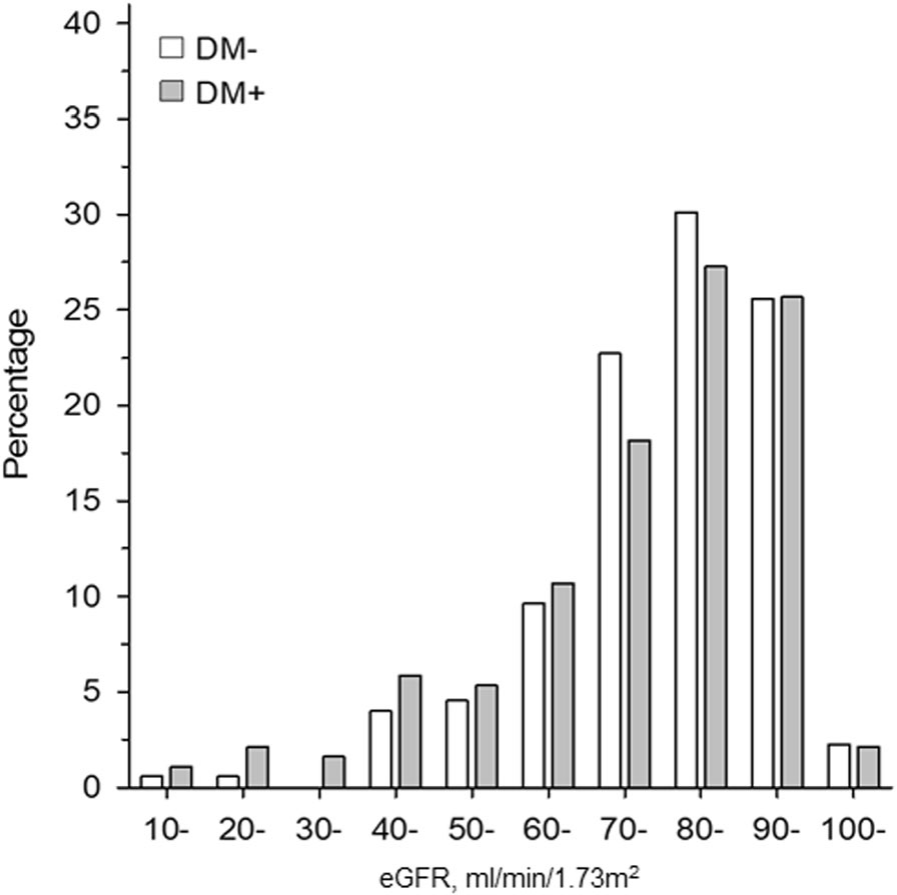
The proportions of the participants with or without diabetes subdivided according to their eGFR levels (CKD-EPI equation, ml/min/1.73 m^2^)

There was no statistically significant difference between the estimated glomerular filtration rates (eGFR) of the two groups. The mean eGFR value using CKD-EPI equation was 80.5 (±14.8) ml/min/1.73m^2^ in the control group and 77.5 (±18.8) ml/min/1.73m^2^ in the group diagnosed with diabetes (*p* = 0.089). The number of the participants who had eGFR less than 60 ml/min/1.73m^2^ was higher in the group diagnosed with diabetes (*n* = 30, 16%) than in the control group (*n* = 17, 10%), but the difference was not quite statistically significant (*p* = 0.070). The proportions of the participants in different eGFR levels with or without diabetes are presented in Fig. 1. In additional analyze using Cockcroft-Gault equation for eGFR, the mean eGFR for persons with diabetes 90.1 mL/min (SD 35.5) and for persons without diabetes 83.3 mL/min (SD 27.2) (*p* = 0.042).

The mean number of potentially nephrotoxic drugs for each participant was approximately 1.06 (±0.88, *n* = 187) in the group diagnosed with diabetes and 0.97 (±1.05, *n* = 176) in the control group (*p* = 0.39). Each participant was categorized into a class (A, B, C or D) based on the nephrotoxic profile of their medications by using the Pharao® database (Table 3). In both groups, more than 90% of the participants were placed into class A, which designates no increased risk for nephrotoxicity. Four participants (1.11%) were in the classes C and D, which indicates either a moderately or a highly increased risk for nephrotoxicity. Three of them were in the class C, two with and one without diabetes. One person without diabetes was categorized into the class D. The mean eGFR values were calculated for each class A to D based on categorized participants. In class A, the mean eGFR value using CKD-EPI equation was 79.1 (±16.8, *n* = 334) ml/min/1.73m^2^. In the classes B, C and D, the mean eGFR values were 77.6 (±18.4, *n* = 25), 82.4 (±28.2, n = 3) and 62.9 (*n* = 1) ml/min/1.73m^2^, respectively.

**Table 3:** Number of participants in the different nephrotoxicity classes

Nephrotoxicity	Number of participants		Total *n* (%)
	No diabetes *n* (%)	Diabetes *n* (%)	
A	162 (92.05)	172 (91.98)	334 (92.01)
B	12 (6.82)	13 (6.95)	25 (6.89)
C	1 (0.57)	2 (1.07)	3 (0.83)
D	1 (0.57)	0 (0.00)	1 (0.28)
Total	176 (100)	187 (100)	363 (100)

A = No need for dosage modification; B = The information is not available or the recommendation is estimated based on the pharmacokinetic characteristics of the substance; C = Modification of the dose or dosage interval is needed; D = The use should be avoided

## Discussion

Our main findings were that there were no differences in kidney function or in the use of potentially nephrotoxic drugs among older home-dwelling persons with or without diabetes. In those persons diagnosed with diabetes, the mean eGFR value was not significantly lowered nor was there any significant difference in the proportion of people with eGFR less than 60 ml/min/1.73m^2^. In addition, only 1 % of the participants were using drugs which are known to carry a moderately and highly increased risk of nephrotoxicity. In additional analyze using Cockcroft-Gault equation for eGFR, person with diabetes had slightly higher mean eGFR than persons without diabetes. However, the values categorized as normal renal function for both groups.

In previous studies, diabetes has been associated with a higher risk of renal failure and a higher rate of renal function decline [4–8]. Moreover, Polonia et al. [17] stated that a higher proportion of hypertensive persons with diabetes had their eGFR value reduced by more than 10% per year and furthermore, also they displayed elevated incidences of moderate and severe renal failure, in comparison with their hypertensive counterparts without diabetes. In the present study, there was no difference in the proportion of people with eGFR less than 60 ml/min/1.73m^2^ between the groups. In the study conducted by Hobeika et al. [6], persons with diabetes did not differ in their baseline eGFR values when compared to their counterparts without diabetes, which is consistent with the present study. The study population consisted of persons diagnosed with hypertension with a mean age of 69 years in both groups. Contrary to the results of the present study and those of Hobeika et al. [6], Yokoyma et al. [8] reported that participants without diabetes had lower baseline eGFR values. The mean age of their study population was less than 65 years and persons without diabetes had been more frequently diagnosed with hypertension, which could explain the lower baseline eGFR value.

Older persons are more prone to drug-induced renal impairment, because of the age-associated deterioration in kidney function [11–13]. In this study, only one in every hundred participants used drugs associated with a moderately and highly increased risk of nephrotoxicity. In a population-based study conducted by Breton et al. [18], 13.3% of the participants were exposed to drugs which were either contraindicated or would require dosage adjustment, when the kidney function is insufficient. The mean number of nephrotoxic drugs per participant was 1.2 ± 0.6. In the present study, the mean numbers of the potentially nephrotoxic drugs were approximately 1.06 and 0.97 drugs per person with and without diabetes, respectively. In three studies investigating Australian and American community-dwelling older persons and nursing home residents, 6–28% had been prescribed drugs which were either contraindicated or required dosage adjustments due to their kidney function [19–21]. In our previous study, one the most clinically relevant drug-drug interactions concerned NSAID and antihypertensives such as ACE-inhibitors, combination that has also increased risk of nephrotoxicity [30].

The mean number of potentially nephrotoxic drugs and proportion of persons who used contraindicated or dosage adjustment requiring drugs were smaller here than in the previous studies. Due to the database used in this study, the numbers of drugs categorized as being nephrotoxic are probably less when compared to the other publications [18–21]. For example, there is one obvious difference from the report of Breton et al. [18]; the Pharao® database does not classify diuretics, angiotensin-receptor blockers and angiotensin-converting enzyme inhibitors as nephrotoxic drugs. In fact, drugs acting through the renin-angiotensin-aldosterone system have been shown to be renoprotective in adults and are recommended for patients with both diabetes and hypertension [31–34]. However, it should be noted that those studies were not performed in older participants. Due to the lack of evidence and the paucity of studies conducted in older participants, the categorization of nephrotoxic drugs used in the Pharao® database may be viewed as acceptable [35]. Hedna et al. (2019) reported high specificity and consequent usefulness of Pharao® database as screening tool. However, further studies was recommended to further study sensitivity in relation to dosage information [36].

The strengths of this study are its population-based study sample, which characterizes older home-dwelling primary care patients diagnosed with diabetes, along with the high questionnaire response and health examination participation rates. On the other hand, the study population originated from one primary care district and therefore a direct generalization cannot be made at even the national level.

There are some limitations; the study subjects were identified from the electronic patient records three months before the questionnaires were dispatched, thus the study population could not include persons with recent onset of diabetes. Furthermore, due to the cross-sectional nature of this study, we were not able to assess the annual reduction in kidney function. The study population consisted of home-dwelling older persons diagnosed with diabetes. It is conceivable that those persons whose eGFR had declined by more than 10% per year were no longer able to live at home and therefore they could have been under-represented in the present study population. Persons with diabetes usually have regular health care visits to ensure good management of their disease. This can be seen in the results; the participants diagnosed with diabetes had lower blood pressure and better cholesterol values, than persons without diabetes. In the present study, we were not able to include the dosage information and the used databases do not have a possibility for the dosage assessment.

Nevertheless, more studies should be conducted to determine the role of diabetes in the kidney function of older individuals. Changes in kidney function should be measured over time and compared with different age-subgroups. Therefore, large enough sample sizes should be used to able the subgroup analysis. In addition, more population-based studies should be conducted in older persons to determine their exposure to inappropriate drugs with dosage information and association with the declines occurring in kidney function.

## Conclusion

The results of this study indicate that the kidney function of older home-dwelling persons with diabetes does not differ from that of older persons without diabetes and furthermore that use of potentially nephrotoxic drugs play only a minor role in the in the worse renal function of these individuals.

## Supplementary information

**Additional file 1: Table S1.** Nephrotoxic drugs (The Pharmacological Risk Assessment Online system: Pharao®). The Pharmacological Risk Assessment Online system (Pharao®) was used to identify potentially nephrotoxic drugs.

## Data Availability

The dataset used and analyzed during the current study are available from the corresponding author on reasonable request.
