# Supplementary material for: Kidney function and nephrotoxic drug use among older home-dwelling persons with or without diabetes in Finland
Source: BMC Nephrol. 2020 Jan 10;21:11. doi: 10.1186/s12882-020-1684-4 (PMC6954600; doi:10.1186/s12882-020-1684-4)
Supplement: Supplementary file 1 — Additional file 1: Table S1. Nephrotoxic drugs (The Pharmacological Risk Assessment Online system: Pharao®). The Pharmacological Risk Assessment Online system (Pharao®) was used to identify potentially nephrotoxic drugs. [file 12882_2020_1684_MOESM1_ESM.docx]

Supplementary table. Nephrotoxic drugs (The Pharmacological Risk Assessment Online system: Pharao®)

| acetylsalicylic acid | iodixanol | zoledronic acid |
| --- | --- | --- |
| acyclovir | iohexol |  |
| adefovir | iomeprol |  |
| amphotericin B | iopromide |  |
| auranofin | ioversol |  |
| azacitidine | joksaglin acid |  |
| balsalazide | ketoprofen |  |
| bekanamysiini | ketorolac |  |
| carboplatin | lansoprazole |  |
| celecoxib | lithium |  |
| cisplatin | lomustine |  |
| clodronate | mefenamic |  |
| clozapine | meloxicam |  |
| colistin | mesalazine |  |
| cyclosporine | methotrexate |  |
| dapsone | mitomycin |  |
| deferasirox | nabumetone |  |
| deksketoprofen | naproxen |  |
| diatrizoic acid | natriumauratiomalate |  |
| diclofenac | olsalazine |  |
| esomeprazole | omeprazole |  |
| etodolac | palifermin |  |
| etoposide | pamidronate |  |
| etoricoxib | pantoprazole |  |
| fenofibrate | parecoxib |  |
| gadobenic | peginterferonialfa-2b |  |
| gadobutrol | pemetrexed |  |
| gadoksetin acid | rabeprazole |  |
| gadopentetic | rifampicin |  |
| gadoteric acid | sirolimus |  |
| gadoteridol | sulfadiazine |  |
| gadoversetamide | sulfasalazine |  |
| gemcitabine | tacrolimus |  |
| gentamicin | teicoplanin |  |
| ibuprofen | telavancin |  |
| ifosfamide | temsirolimus |  |
| imipenem | tenofovir |  |
| immunoglobulin | tobramycin |  |
| indinavir | tolfenamic |  |
| indomethacin | tretinoin |  |
| interferon alpha | valaciclovir |  |
| interferon beta | vancomycin |  |
